# HERA: a web server for host element reference-based aligner

**DOI:** 10.1093/nar/gkag448

**Published:** 2026-05-14

**Authors:** Leidy-Alejandra G Molano, Pascal Hirsch, Andreas Keller, Monika Dolejska, Jana Palkovicova

**Affiliations:** Chair for Clinical Bioinformatics, Saarland University, Saarbrücken 66123, Germany; Helmholtz Institute for Pharmaceutical Research Saarland, Helmholtz Center for Infection Research, Saarbrücken 66123, Germany; Chair for Clinical Bioinformatics, Saarland University, Saarbrücken 66123, Germany; Chair for Clinical Bioinformatics, Saarland University, Saarbrücken 66123, Germany; Helmholtz Institute for Pharmaceutical Research Saarland, Helmholtz Center for Infection Research, Saarbrücken 66123, Germany; PharmaScienceHub, Saarland University, Saarbrücken 66123, Germany; Department of Biology and Wildlife Diseases, University of Veterinary Sciences Brno, Brno 61242,Czech Republic; Central European Institute of Technology, University of Veterinary Sciences Brno, Brno 61242,Czech Republic; Department of Microbiology, Faculty of Medicine in Pilsen, Charles University, Pilsen 32300,Czech Republic; Division of Clinical Microbiology and Immunology, Department of Laboratory Medicine, University Hospital Brno, Brno 61242,Czech Republic; Department of Chemistry and Biochemistry, Faculty of AgriSciences, Mendel University in Brno, Brno 61242,Czech Republic; Central European Institute of Technology, University of Veterinary Sciences Brno, Brno 61242,Czech Republic; Department of Microbiology, Faculty of Medicine in Pilsen, Charles University, Pilsen 32300,Czech Republic

## Abstract

Plasmids play a central role in bacterial adaptation and in the dissemination of antimicrobial resistance, driving a growing need for accessible tools that support their comparative analysis without requiring local computational infrastructure. Although several circular genome visualization platforms exist, most are designed for general bacterial genome analysis rather than focused on plasmid comparison. Host element reference-based aligner (HERA) is a web server for intuitive visualization and comparison of plasmids and other circular molecules through BLAST alignment against reference sequences. Built on interactive circular genome visualization, HERA simplifies comparative genomics by providing an accessible interface for exploring sequence similarity, identifying conserved regions, and analyzing genetic elements without the complexity of traditional local tools. HERA includes a plasmid-oriented annotation pipeline covering replicon and mobility typing, antimicrobial resistance detection, mobile element identification, and homology search against the PLSDB plasmid database. HERA also provides an automatic selection of the reference which is the most appropriate from the uploaded sequences. The web server is available without login or any restriction at https://web.ccb.uni-saarland.de/hera/.

## Graphical Abstract

**Graphical Abstract:**
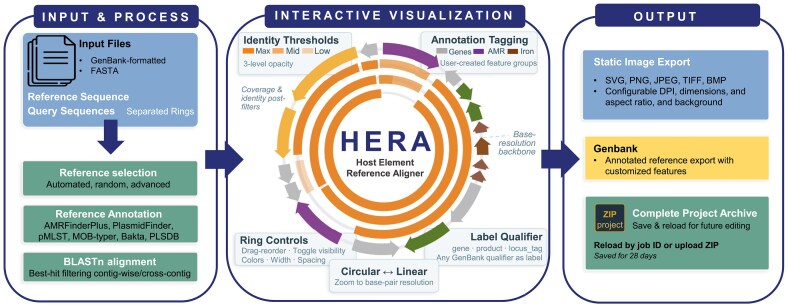

## Introduction

In the era of genomic data, thousands of prokaryotic genome sequences are now available in public databases [1, 2]. Among these, plasmids and other mobile genetic elements are of particular interest due to their role in antimicrobial resistance dissemination and bacterial adaptation [3–5]. Effective visualization of these circular molecules is essential for interpreting comparative genomics results, identifying conserved regions, and detecting gene gain and loss events.

Due to the inherent difficulty of rendering circular images in common visualization packages (i.e. ggplot package), several tools have been developed, each with distinct capabilities and limitations. Circos is a very flexible framework capable of rendering complex multi-track plots with ribbon and link diagrams connecting genomic positions [6]. However, it is Perl-dependent and offers no graphical user interface. GenoVi attempted to improve its accessibility by creating a Python command-line wrapper around Circos [7], automating the creation of circular genome representations from GenBank input files. However, both Circos and GenoVi are limited to static image generators. The CGView family of tools extended these capabilities by allowing BLAST-based comparisons of a reference genome against other sequences with automatic Clusters of Orthologous Genes classification and base composition plots [8, 9]. However, like Circos and Genovi, GCView Comparison Tool is command-line-driven, produces only static images, and requires installation of multiple dependencies including Perl, Java, and BLAST+ . BRIG (BLAST Ring Image Generator) addressed the usability challenge by providing a cross-platform Java desktop application with a graphical user interface for generating circular BLAST comparison images [10, 11]. Despite its popularity, BRIG produces also only static images without interactive exploration capabilities. More recent web-based tools have expanded the landscape of circular genome visualization, such as Proksee, a comprehensive web server that integrates genome assembly, annotation, and visualization into a unified platform, generating interactive maps powered by CGView.js [5, 12]. While Proksee represents the most feature-rich option currently available, its BLAST comparison functionality is limited to approximately five comparison genomes, and it is primarily oriented toward comprehensive bacterial genome analysis rather than plasmid comparison.

To address these gaps, we developed HERA (host element reference-based aligner), a web server that combines the analytical power of BLAST-based multi-sequence comparison with an intuitive, highly customizable web interface for interactive circular genome visualization. HERA is designed for comparing plasmids and contig-based genome assemblies against a reference sequence, providing researchers with an accessible platform that requires no software installation, no programming knowledge, and no computational infrastructure.

## Materials and methods

HERA is implemented as a Django 5.2 web application with a modular architecture composed of three main components. First, the initial submission interface, where users are prompted to upload the sequences. Second, our server-side data pipeline to align the plasmids and generate the plot data. Lastly, the result interface offers interactive data exploration, optimization of plot features, and exporting capabilities.

The web application is served via a containerized deployment with an allocated capacity of 16 CPU cores and 32 GB of RAM. Asynchronous analysis jobs are managed via a Celery task queue, configured with four workers, allowing up to four concurrent submissions to be processed simultaneously. Upload file sizes are currently limited to 120 MB per file.

### Reference and query selection

HERA accepts GenBank-formatted or FASTA files as reference and query sequences. Reference selection can be provided by the user or delegated to HERA via three modes: fully automatic, random, or advanced (user-defined length and alignment scoring policies). In fully automatic mode, HERA evaluates all uploaded single-contig files as candidate references and selects the one that best represents the dataset. Each candidate is scored on two independent criteria: sequence length and pairwise alignment similarity.

The length score rewards longer assemblies, under the assumption that more complete sequences provide a better reference backbone. Scores are normalized so that the longest candidate receives the maximum value and the others are scaled proportionally.

The alignment score captures how well each candidate represents the rest of the dataset. For each candidate, a local BLAST database is built and all other uploaded sequences are aligned against it using BLASTn. The resulting query coverage and percentage identity values are averaged per candidate, normalized across candidates, and combined with equal weighting. If no alignments are produced, all candidates receive a score of zero for that metric.

The final score is the mean of the length and alignment scores, giving equal importance to assembly completeness and sequence representativeness. The highest-scoring candidate is selected as reference, with ties broken by upload order, and the remaining files are used as queries.

### BLAST

Upon submission, the selected reference file is used to construct a local BLAST database (v. 2.17.0) [11], which is used to align the queries using the BLASTn algorithm. BLAST results undergo a cross-contig best-hit filtering step based on the NCBI Best-Hits filtering algorithm. This filter removes redundant HSPs (high-scoring segment pairs) from different contigs of the same query file using three criteria: (i) the *e*-value of HSP A must be greater than or equal to that of HSP B; (ii) the bit score density of A must be strictly lower than that of B by a configurable score-edge factor; and (iii) the subject regions of A and B must overlap, with A extending beyond B by no more than a configurable overhang fraction of B’s subject length.

### Automated annotation of reference

The reference sequence is processed through an asynchronous Snakemake pipeline (v 9.19.0) [13] comprising: PLSDB (BLASTn search, database v.2024_05_31_v2) [14], AMRFinderPlus (v. 4.0.19, database v.2025-03-25.1) [15], PlasmidFinder (v. 2.1.6, database v.2.2.0) [16], pMLST (v. 2.23.0, database download on 2025-06-14) [17], MOB-typer (v.3.1.9, database v.3.1.8,) [18], and Bakta (v.1.12.0, database v6) [19].

### Visualization

Upon completion of BLAST alignments, HERA generates an interactive circular map powered by CGView.js (v.1.8.0), augmented with D3.js (v.7) for custom legend rendering, jsPanel (v. 4.15.0,) for draggable control windows, and DataTables (v.2.3.4) for interactive tabular data management. Image exporting is supported by html2canvas (v.1.4.1).

## Results

### Automatic reference selection and annotation

HERA provides three reference selection modes: (i) In automatic mode, all uploaded sequences are scored based on length metrics and pairwise BLAST alignment to select the optimal reference. (ii) A random mode is available for exploratory use. (iii) An advanced mode allows users to define their own length policy (longest, shortest, or closest to average) and alignment scoring policy (coverage, similarity, balanced, or ignored).

HERA also allows an extended annotation pipeline that automatically annotated selected the reference sequence upon submission. Users can select their analysis of interest and specify their own parameters. Currently, the extended analysis options are: PLSDB (BLASTn search), AMRFinderPlus, PlasmidFinder, pMLST, MOB-typer, and Bakta. Results are displayed in a dedicated “Extended Analysis” panel with searchable and sortable DataTables. All annotated features are automatically integrated into the CGView circular map and are subject to the same interactive visibility and labeling controls available for user-defined annotations.

### Interactive control panels

HERA provides real-time customization through independent draggable control panels: HERA Controls, Tracks Data, and Extended Analysis (if automatic reference annotation was selected). HERA Controls represents the main control panel, organized into tabbed sections for General settings and Export options. The General tab provides controls for identity thresholds, ring display properties, map format switching between circular and linear views, reference title customization, query title positioning and styling, backbone appearance, tick mark properties, and annotation display options. The Export tab provides controls for saving and downloading session data, as well as a comprehensive image export interface.

The second control panel (Tracks Data) provides three tabbed views: Samples, Annotations, and BLAST. The Samples tab presents an interactive table listing all query rings, where users can reorder rings via drag-and-drop, toggle individual ring and label visibility. The Annotations tab displays all reference annotation features in a searchable and selectable table and provides an annotation grouping system where users can create named tag groups, assign selected features to groups, and automatically generate color-coded legend entries for each group. This annotation tagging system allows users to highlight important genetic features such as resistance genes, virulence factors, or other genetic elements in distinct colors. The BLAST tab presents all BLAST hits in a filterable table with adjustable minimum thresholds for query coverage, subject coverage, percentage identity, and alignment length.

The third panel (Extended Analysis) displays the results of the automated reference annotation pipeline. When extended analysis tools are selected, the panel provides one tab per tool, each presenting results in a searchable and sortable table.

Three interactive legends are displayed directly on the map: an identity opacity legend, a layout legend displaying query ring assignments, and an annotation tags legend showing user-created feature groups. All three legends are independently toggleable and draggable.

### Export and session management

Session data can be saved to the server for later retrieval (with 28-day retention) or downloaded as a ZIP archive containing the CGView JSON configuration, session state JSON, original GenBank and FASTA files, and all BLAST alignment results. Additionally, tables in CSV/TSV formats for reference annotation and selection scoring will be included if these options were selected. Saved sessions can be restored either by entering the job ID on the Results page or by uploading a previously downloaded ZIP file, which restores the complete visualization state including all customizations. The ZIP file incorporates a README.md file that includes all tools specifications to ensure reproducibility.

HERA supports image export in five formats: SVG for scalable vector graphics, PNG, JPEG, TIFF, and BMP for raster formats. The export interface provides configurable dimensions, adjustable DPI, aspect ratio locking, transparent background option, custom cropping, and a live image preview.

The complete annotated reference reflecting the current map state, including extended analysis features, can be exported in GenBank format.

### Use case: comparative analysis of antimicrobial resistance plasmids

**Figure 1.**
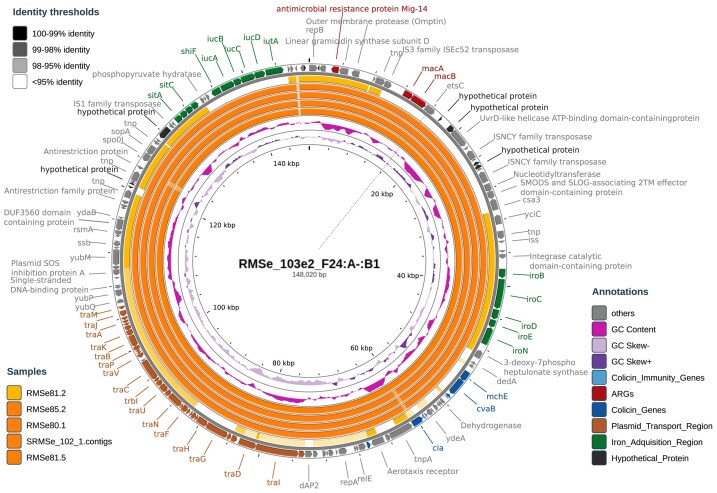
Circular comparison of F24:A-:B1 plasmids from *E. coli* ST11138 and ST616 isolates generated using HERA. The F24:A-:B1 plasmid from isolate RMSe 103e2 (ST11138) was used as the reference sequence (outermost annotation ring). Each concentric ring represents a query plasmid aligned by BLASTn, with rings ordered by decreasing similarity to the reference. BLAST hits are colored by a three-level opacity system reflecting percentage identity thresholds set at 99%, 98%, and 95%. Annotated features on the reference ring are grouped into user-defined categories: antimicrobial resistance genes, colicin and colicin immunity genes, iron acquisition genes, and plasmid transfer region. GC content and GC skew are displayed as the innermost tracks. Original plasmid data were published in Ruzickova *et al*. [20].

To illustrate HERA’s capabilities (Fig. 1), we analyzed IncF-type plasmids carrying antimicrobial resistance and colicin genes from cephalosporin-resistant *Escherichia coli* isolated from Caspian gulls, as described by Ruzickova *et al*. [20]. The IncF replicon sequencing type (RST) F24:A-:B1 plasmid corresponding to *E. coli* ST11138 (isolate RMSe 103e2), was uploaded as the GenBank-formatted reference. Five query plasmid sequences from the same F24:A-:B1 RST, representing two *E. coli* sequence types, were uploaded as FASTA files: ST616 was represented by RMSe81.2, whereas ST11138 was represented by RMSe85.2, RMSe80.1, SRMSe.102.1, and RMSe81.5. The resulting circular map displayed the reference backbone with annotated CDS features, with each query plasmid rendered as a separate concentric ring. Identity thresholds were adjusted to 99%, 98%, and 95% using the HERA Controls panel to resolve fine-scale differences among these closely related plasmids. The annotation tagging system was then used to create custom feature groups highlighting antimicrobial resistance genes, colicin and colicin immunity genes, iron acquisition genes, and plasmid transfer regions, each displayed in a distinct color on the reference ring. Ring order was adjusted to place the most dissimilar plasmid (RMSe81.2, ST616) adjacent to the reference for clearer visual contrast.

The HERA visualization revealed several biologically informative patterns. The plasmids from the four ST11138 isolates showed near-complete coverage at the highest identity level. In contrast, the same F24:A-:B1 RST from ST616 isolates displayed visible gaps in the colicin-producing operon region, including the *cvaC* colicin gene, *cvaB*, and *mchE*, as well as differences in the antimicrobial resistance genes *macA* and *macB*. This presumably explained the domination of ST11138 over other STs.

## Discussion

HERA addresses a specific gap in the landscape of circular genome visualization tools: the need for an accessible, web-based platform that combines BLAST-based multi-sequence comparison with extensive real-time interactive customization specifically oriented toward plasmid analysis. While existing tools require either command-line expertise (CCT, Circos, GenoVi), desktop software installation (BRIG), or are designed for broader purposes (Proksee), HERA provides a focused and intuitive workflow for comparison of assembled plasmids or contig-based genome assemblies against a selected reference sequence, with visualization optimized for circular maps but adaptable to linear layouts.

While HERA intentionally builds on established, well-validated tools such as BLAST+ for sequence alignment and CGView.js for circular rendering, its main contribution lies in their integration with features not previously combined in a single accessible platform. Several features distinguish HERA from other existing tools (Table 1). First, HERA’s annotation tagging system allows users to create custom feature groups and assign reference annotations to visually distinct categories. This is particularly valuable for highlighting functionally relevant genomic features such as antimicrobial resistance determinants or virulence factors. Second, the draggable panel-based interface allows users to position controls alongside the visualization without obscuring the map, unlike fixed sidebar approaches. Third, HERA implements a best-hit filtering algorithm adapted from the NCBI specification that reduces redundant HSPs before visualization, and provides post-hoc filtering by query coverage, subject coverage, identity, and alignment length in the interactive BLAST table.

**Table 1.** Feature comparison of circular genome visualization tools

Feature	HERA	BRIG	Proksee	CGView CT	Circos
Web-based	✓	✗	✓	✗	✗
Automatic reference selection	✓	✗	✗	✗	✗
BLAST-based comparison	✓	✓	✓	✓	✗
Best-hit HSP filtering	✓	✗	✗	✗	-
AMR detection	✓	✗	✓	✗	✗
Plasmid typing (replicon, MOB, MLST)	✓	✗	✗	✗	✗
IS element / transposon annotation	✓	✗	✗	✗	✗
Structural & functional annotation (CDS, tRNA, ncRNA)	✓	✗	✓	✗	✗
Plasmid homology search (PLSDB)	✓	✗	✗	✗	✗
Interactive visualization	✓	✗	✓	✗	✗
Real-time adjustments	✓	✗	✗	✗	✗
Annotation grouping/tagging	✓	✗	✗	✗	✗
Session save/restore	✓	✗	✓	✗	✗
GenBank export of reference sequence	✓	✗	✗	✗	✗
Multi-format image export	✓	✓	✓	✓	✓

HERA’s visualization is built upon CGView.js and inherits its constraints, including a maximum sequence capacity of 10 Mb and the inability to display structural links between query and reference sequences. Currently, only BLAST is available for sequence comparison; alternative alignment methods such as minimap2 [21] are not yet supported. Additionally, HERA operates on a single-reference paradigm and does not offer clustering-based approaches for grouping related sequences or selecting representative references from large collections. Finally, support for long-read data through Mash-based distance estimation, multi-reference comparison workflows, and automatic identification of plasmid-associated contigs from draft genome assemblies, represent further directions for future development.

## Data Availability

HERA is freely available without any login requirement at https://web.ccb.uni-saarland.de/hera/.
